# Morphomolecular characterization and statistical assessment of environmental associations with *Diplectanum* infestation in cultured European sea bass and bath treatment efficacy

**DOI:** 10.1038/s41598-026-63796-2

**Published:** 2026-07-30

**Authors:** Amr Fadel, Ahmed Fathy, Mahmoud Mabrok, Mohamed Bessat

**Affiliations:** 1 National Institute of Oceanography and Fisheries (NIOF), Cairo, Egypt; 2 Department of Animal Wealth Development, Biostatistics Division, Faculty of Veterinary Medicine, Suez Canal University, Ismailia, 41522 Egypt; 3 Department of Aquatic Animal Medicine, Faculty of Veterinary Medicine, Suez Canal University, Ismailia, 41522 Egypt; 4 Department of Parasitology, Faculty of Veterinary Medicine, Alexandria University, Alexandria, Egypt; 5 Faculty of Veterinary Medicine, King Salman International University, South Sinai, Egypt

**Keywords:** *Dicentrarchus labrax*, Monogenean prevalence, Phylogeny, Multivariate environmental analysis, *Yucca schidigera*, Fenbendazole, Ecology, Ecology, Microbiology, Zoology

## Abstract

Monogenean infections are one of the most economically important parasitic constraints in marine aquaculture; however, knowledge remains limited regarding their genetic diversity, epidemiology, and sustainable management strategies. This study investigated the prevalence, morphomolecular characterization, environmental drivers, and eco-friendly control of naturally occurring monogenean infections in European sea bass (*Dicentrarchus labrax*) from two marine farms along the Egyptian Mediterranean coast between February and May 2024. Infected fish exhibited respiratory distress, skin darkening, anorexia, and progressive emaciation under heavy infestation. Examination of gill smears identified an elongate-oval monogenean exhibiting morphological features consistent with *D. laubieri*. Phylogenetic analysis of partial *18 S rRNA* gene sequences placed the specimen within the *Diplectanum* clade, corroborating its taxonomic affiliation (GenBank accession no. PZ253293.1). Parasitological indices varied significantly between farms, with a higher prevalence recorded at Farm 2 (75.00 ± 6.87%) than at Farm 1 (65.56 ± 7.51%) (*p* < 0.05). Seasonally, the highest prevalence occurred in February, followed by a decline in April and a slight increase in May. Water quality analysis indicated a progressive rise in temperature on both farms, reaching 25.40 ± 0.16 °C in May. In contrast, Farm 2 was characterized by higher salinity, pH, and total ammonia levels, together with lower dissolved oxygen concentrations. Correlation analysis demonstrated a strong negative association between parasite prevalence and water temperature (*r* = − 0.736, *p* < 0.05), whereas no significant correlations were observed with salinity, pH, dissolved oxygen, or total ammonia. In trials of treating the affected fish and based on toxicity testing, the safe exposure threshold for *Y. schidigera* extract was established at 150–200 µg/L, while fenbendazole exhibited a safe exposure limit of 20 µg/L. The experimental cohabitation successfully reproduced the infection, achieving a prevalence of 90% by day 14. Comparative therapeutic efficacy trials demonstrated the superior performance of *Y. schidigera* relative to fenbendazole under the present study conditions, with parasite reduction rates of 80.4% and 68.5%, respectively, accompanied by a lower incidence of mortality. Collectively, these findings highlight the combined influence of environmental conditions on monogenean infection dynamics and support phytogenic-based *Y. schidigera* as a promising eco-friendly alternative for parasite control in marine aquaculture systems.

## Introduction

Mariculture has emerged as one of the fastest-growing sectors supplying animal protein worldwide, playing a vital role in meeting the rising demand for food security^1^. Despite this rapid expansion, the sustainability of fish farming remains challenged by a complex mix of economic pressures, environmental constraints, and disease outbreaks^2,3^. Infectious diseases remain a dominant limiting factor, with pathogens of bacterial, viral, and parasitic origin posing persistent threats to productivity and profitability^4–6^.

Monogenean helminths constitute a major subgroup of Trematoda with a direct monoxenous life cycle infecting fish, posing a significant health threat and limiting aquaculture productivity. They are broadly classified into two primary groups: Polyopisthocotylea and the species-rich subclass Monopisthocotylea^7,8^. Within the Monopisthocotylea, the family Diplectanidae comprises important parasitic species, notably *Diplectanum aequans*^9^ and *Diplectanum laubieri*^10^, both of which have been well documented in earlier studies^11,12^. These parasites are widely distributed across freshwater, marine, and brackish habitats, where they infest fish and, in some cases, invertebrate hosts^8^. Monopisthocotylean monogeneans are typically found on external surfaces such as the skin and gills. Moderate to severe infestation with these helminths often leads to irritation, excessive mucus production, tissue opacity, localized redness, and hemorrhagic lesions^13^. In European sea bass, outbreaks of *Diplectanum* species have been reported globally in both farmed and wild populations, frequently resulting in substantial economic losses^14–16^.

The classification of monogeneans of the genus *Diplectanum* has traditionally been based on morphological features, particularly haptoral and male copulatory structures^17^. However, morphological similarities often limit accurate discrimination among closely related species. Consequently, molecular approaches, including ribosomal and mitochondrial DNA sequencing, have increasingly been applied to improve species delimitation, clarify phylogenetic relationships, and define population genetic structure within Diplectanidae^18,19^. Recent studies have demonstrated the utility of ITS rDNA and 28 S rDNA markers for distinguishing cryptic species and reconstructing phylogenetic relationships among *Diplectanum* and related diplectanid taxa^20,21^.

Resistance development, host toxicity, environmental impacts, and regulatory restrictions on residues increasingly limit conventional chemotherapeutics for the control of monogenean infection^22^. These constraints have accelerated interest in phytobiotic alternatives, which offer antiparasitic, immunostimulatory, and biodegradable properties with reduced ecological risk^23–26^. Modern aquaculture prioritizes eco-friendly, integrated preventive strategies that reduce pathogen emergence and reinfection while enhancing system sustainability^27–30^.

*Yucca schidigera* is a perennial xerophytic plant of the family Asparagaceae that is native to arid regions of the southwestern United States and northern Mexico^31^. This species has also been introduced and adapted for cultivation in arid and semiarid regions, including Egypt, where it is used in agricultural and aquaculture systems as a feed and water-quality additive under desert environmental conditions^32^. Dietary supplementation with *Y. schidigera* has been widely used among various cultured fishes as a functional additive for improving growth performance, feed utilization, water quality, antioxidant status, immune response, and disease resistance, largely because of its steroidal saponins, polyphenols, and resveratrol^33^. Beneficial immunostimulatory and water-quality–improving effects of *Y. schidigera* supplementation have been reported in European sea bass (*D. labrax*), Nile tilapia (*Oreochromis niloticus*), common carp (*Cyprinus carpio*), and whiteleg shrimp (*Litopenaeus vannamei*)^34–36^. However, existing studies have focused mainly on dietary supplementation and water quality improvement, with limited evidence on their therapeutic use for the treatment of established parasitic infections.

Despite the pathogenic significance of monogenean infections, limited information is available on the taxonomy, genetic diversity, and effective control of *Diplectanum* spp., particularly regarding eco-friendly and sustainable approaches. Therefore, this study aimed to investigate the parasitological characteristics and molecular identification of *Diplectanum* spp. infecting cultured *D. labrax*. Additionally, infection prevalence, parasite intensity, and mortality rates across the study period and locations were assessed in relation to physicochemical water parameters. Furthermore, this study evaluated the comparative therapeutic efficacy and safety of *Y. schidigera* and fenbendazole bath treatments following experimental monogenean infection.

## Materials and methods

### Study locations and fish sampling

A total of 360 European sea bass, *Dicentrarchus labrax*, were randomly sampled from two earthen pond farms (*n* = 180 fish per farm). Farms were located in geographically distinct regions of Egypt, with Farm 1 located in Borg El Arab, Alexandria Governorate (30°50′56″ N, 29°36′42″ E), whereas Farm 2 was situated in Edeeba, Shata, Damietta Governorate (31°24′32″ N, 31°52′19″ E). At the time of sample collection, both farms were experiencing disease outbreaks. Fish were collected monthly over four months from February to May 2024, with 45 fish sampled per month from each farm. All the sampled fish underwent clinical examination in accordance with standard diagnostic procedures^37,38^. Clinical assessment included the observation of abnormalities in swimming behavior, respiratory distress, feeding activity, and external abnormalities such as skin discoloration, excessive mucus secretion, erosions, hemorrhages, ulcers, and fin erosion.

### Physicochemical water examination

Basic physicochemical water quality parameters, including temperature, dissolved oxygen, pH, and salinity, were measured in situ using a multiparameter electronic probe in accordance with APHA guidelines^39^. In addition, 5-L water samples were collected from the same ponds at a depth of 0.5 m via a horizontal water sampler for analysis of total ammonia (NH₄⁺). The samples were stored in clean glass bottles, transported in insulated coolers to the laboratory, and analyzed according to the Discharge Standards of Marine Aquaculture Water (DB 21/T 2428, 2015) and following previously described protocols^40–42^.

### Parasitological examination

Scrapings of gill filaments were carefully examined under a dissecting microscope (Optika, Italy) for the detection and collection of parasites. Monogenean parasites were recovered from the gills via wet mount preparations. The collected material was transferred onto clean glass slides containing a drop of physiological saline, gently dispersed to facilitate parasite release, and covered with a coverslip. Fresh preparations were examined immediately under a light microscope at different magnifications for the detection and morphological identification of isolated monogeneans. For detailed morphological analysis, individual parasites were assembled via micropipettes and fine dissecting needles and were transferred to a fixative solution. For the detailed morphometric examination, some of the samples were fixed in 5% buffered formalin saline to maintain morphological integrity, whereas the remaining samples were preserved in 70% ethanol at -20 °C for long-term storage and subsequent molecular analyses. Before identification, selected specimens were prepared for morphological examination by applying gentle coverslip pressure to achieve proper flattening, enabling improved visualization of diagnostic features such as the haptor and reproductive structures. Taxonomic identification and classification were performed based on morphometric criteria following established diagnostic keys and descriptions by Whittington^43^.

### Molecular identification

Molecular characterization of the isolated diplectanid monogeneans was performed to confirm genus/species validity, following the molecular sequencing protocol of Porter et al.^44^, with some modifications. For the molecular analysis, representative isolates were selected from samples initially identified via microscopy as *Diplectanum laubieri*. The selected samples were removed from alcohol and air-dried before total genomic DNA extraction was conducted via a DNeasy kit (Qiagen). The primer pair WormA (GCGAATGGCTCATTAAATCAG) and WormB (CTTGTTACGACTTTTACTTCC), which target a partial region of the nuclear *18 S rRNA* gene, was used for PCR amplification and subsequent phylogenetic analysis. PCR amplification was executed in 25 µL reaction volumes comprising 2 µL of extracted DNA, 10 pM of each PCR primer, and Ready-To-Go PCR beads (Amersham Pharmacia Biotech), each containing 1.5 U Taq polymerase, 10 mM Tris-HCl at pH 9, 50 mM KCl, 1.5 mM MgCl2, 200 µM each dNTP, and stabilizers, including bovine serum albumin. The thermal cycling conditions included initial denaturation at 94 °C for 3 min, followed by 35 cycles of denaturation at 94 °C, annealing at 56 °C, and extension at 72 °C, with a final 10-min extension. The PCR products were resolved by electrophoresis on 1.5% agarose gels containing ethidium bromide at 80 V for 45 min. The bands were visualized under UV light and confirmed by comparison with a DNA molecular weight ladder to ensure the presence of amplicons of the expected size. Gel slices corresponding to the correctly sized amplicons were carefully excised under sterile conditions, subjected to purification via a standard gel extraction protocol, and subsequently subjected to Sanger sequencing to facilitate downstream phylogenetic analysis.

### Sequencing and phylogenetic reconstruction

The eluted and purified PCR-amplified amplicons were processed by Sanger sequencing, using the same primer sets that were used during the initial PCR. The resulting forward and reverse AB1 traces were manually assessed, and the low-quality 3′ and 5′ terminal regions were trimmed. The resulting clean sequences were BLAST-analyzed at the NCBI nucleotide BLAST portal, applying the default settings. Homologous sequences were retrieved from GenBank, and sequence alignment was performed via the CLUSTAL algorithm within MEGA version 11, with subsequent refinements made through manual adjustment^45^. To construct the phylogenetic tree, the aligned 18 S and other homologous nuclear and ITS sequences were analyzed via the neighbor‒joining (NJ) method based on the Tamura‒Nei model within MEGA 11^46,47^. The robustness and reliability of the resulting NJ tree were validated through bootstrap analysis involving 1000 replicates.

## Evaluation of the antiparasitic efficacy of *Y. schidigera* and Fenbendazole against monogenean challenge

### Herbal extract preparation

Fresh *Y. schidigera* plant material was obtained from the Department of Botany, Faculty of Agriculture, Zagazig University. The collected material was thoroughly rinsed with distilled water to remove adhering debris and surface impurities, and then oven-dried at 40 °C for 3 days until a constant weight was achieved. The dried plant material was subsequently milled into a fine powder via a sterile electric grinder and stored in airtight amber glass containers at 4 °C to protect it from light and moisture until further processing. The aqueous–alcoholic extract of *Y. schidigera* was prepared according to the method described by Góngora-Chi^48^, with minor modifications. In brief, 100 g of the powdered plant material was extracted with 1 L of 70% ethanol (1:10, w/v) under controlled ambient conditions. The mixture was subjected to ultrasonic-assisted extraction in an ultrasonic bath (40 kHz; Skymen Cleaning Equipment Shenzhen Co., Ltd.) maintained at 45 °C for 30 min to increase compound release, followed by continuous orbital shaking at 150 rpm for 24 h in complete darkness to prevent the photodegradation of bioactive constituents. The extract was then filtered through Whatman No. 1 filter paper (11 μm pore size) to remove particulate matter. The resulting filtrate was concentrated under reduced pressure at 45 °C via a rotary evaporator, and the semisolid residue was further dried in a vacuum oven at 40 °C to obtain a stable crude extract. The final extract was stored at − 20 °C until subsequent analyses were performed.

### Determination of total phenolic and flavonoid contents

The total phenolic content (TPC) and total flavonoid content (TFC) of the *Y. schidigera* extract were determined via established colorimetric methods with slight modifications. TPC was assessed according to the Folin–Ciocalteu method^49^. Briefly, 0.5 mL of extract was mixed with 2.5 mL of 10% (v/v) Folin–Ciocalteu reagent and allowed to react for 5 min at room temperature, after which 2.0 mL of 7.5% (w/v) sodium carbonate (Na₂CO₃) solution was added. The mixture was incubated in the dark at ambient temperature for 30–60 min to develop color, and the absorbance was measured at 760–765 nm via a UV–visible spectrophotometer (Shimadzu, Kyoto, Japan). The TPC was quantified via a gallic acid standard calibration curve and expressed as milligrams of gallic acid equivalents per gram of dry extract (mg GAE/g DE).

The TFC was determined via an aluminum chloride (AlCl₃) colorimetric assay^50,51^. In brief, 0.5 mL of the extract was combined with 1.5 mL of methanol, followed by the sequential addition of 0.1 mL of 10% (w/v) aluminum chloride (AlCl₃), 0.1 mL of 1 M potassium acetate, and 2.8 mL of distilled water. The reaction mixture was incubated at room temperature for 30 min in the dark, and the absorbance was measured at 415 nm. The flavonoid content was calculated via a quercetin standard calibration curve and expressed as milligrams of quercetin equivalents per gram of dry extract (mg QE/g DE).

### Preparation of *Y. schidigera* extract and fenbendazole for toxicity and therapeutic assays

Dried *Y. schidigera* extract was prepared for water-bath administration following previously described procedures with minor modifications^52,53^. A stock solution (100 g/L) was prepared by dissolving the dried extract in dimethyl sulfoxide (DMSO) so that the final solvent concentration did not exceed 0.1% (v/v). The solution was subsequently diluted with sterile distilled water and homogenized via continuous magnetic stirring for 20–30 min to achieve complete solubilization. The resulting stock solution was freshly diluted with aerated seawater immediately before application in toxicity and therapeutic assays to ensure the stability and consistency of exposure. Fenbendazole was prepared for the experimental assays following established protocols^54–56^. A commercial water-soluble formulation of Fenbendazole (10% w/v; Al Nasr Pharmaceutical Chemicals Co., Egypt) was employed. The required volume was first prediluted in a small aliquot of aerated seawater and then introduced into the experimental system under continuous aeration to ensure uniform dispersion in order to obtain the target treatment concentrations.

### Acclimation conditions and experimental setup

A total of 900 *D. labrax* juveniles (73.58 ± 4.57 g) were collected from a private hatchery with no recorded history of disease outbreaks or production disorders. The fish were acclimated for 14 days in a 5,000 L fiberglass tank supplied with aerated and filtered seawater under continuous aeration and a 12 h light:12 h dark photoperiod^57^. Tank hygiene was maintained via a flow-through system combined with high-efficiency mechanical filtration and daily siphoning of waste. Throughout acclimation and the experimental period, the water quality was maintained within optimal ranges for *D. labrax* (temperature: 27.2 ± 0.65 °C; salinity: 33 ± 2.16‰; dissolved oxygen: 6.24 ± 0.92 mg/L; pH: 7.5 ± 0.46), with 20–30% daily water renewal. The fish were fed a commercial marine diet containing 45% crude protein at 3% body weight twice daily, and feeding was withheld 24 h before exposure to standardize physiological status and minimize metabolic waste.

The operationally safe concentration of *Y. schidigera* extract for bath application under the present experimental conditions was determined according to standard fish toxicity testing protocols, with minor modifications^58^. A 24-hour immersion assay was conducted using serial concentrations of the standardized extract. A total of 180 acclimated fish were randomly allocated into six experimental groups in triplicate (T1–T6; *n* = 3) and maintained in 180 L fiberglass tanks. The fish in T1 served as the untreated control, while those in groups T2–T6 were exposed to graded concentrations of *Y. schidigera* extract (50, 100, 200, 300, and 400 µg/L) prepared from a standardized stock solution.

In parallel, a fenbendazole bath assay was performed using a commercial formulation (50 mg/mL; 10%). Serial dilutions were prepared in seawater to achieve final concentrations of 5, 10, 15, 20, 25, and 30 µg/L^59^. The fish were distributed into seven groups in triplicate (10 fish per tank), including an untreated control (T1) and six treated groups (T2–T7), and exposed for 24 h under continuous gentle aeration. During exposure, fish were continuously monitored for mortality and clinical signs of toxicity. The lethal concentrations were estimated based on cumulative mortality recorded within 24 h post-exposure^60,61^. The operational safe concentration under the present experimental conditions was defined as the highest tested dose that produced no mortality or observable behavioral or clinical signs of toxicity throughout the exposure period.

### Experimental cohabitation parasitic challenge

A cohabitation model was employed to establish experimental monogenean infection in *D. labrax* following established protocols for natural transmission and host–parasite interactions^62^. Fish exhibiting heavy monogenean infestations, as confirmed by microscopic examination of fresh gill wet mounts, were selected as donor fish. In contrast, recipient fish were screened before exposure to ensure that they were free of parasitic infection. For transmission, infected donor fish were cohabitated with parasite-free recipients at a donor-to-recipient ratio of 1:2 for 14 consecutive days, allowing natural parasite dissemination through direct host contact and waterborne infective stages (oncomiracidia). Thereafter, the donor and recipient fish were maintained in a 3-ton tank divided by a nylon hapa net partition (5 mm mesh size), which prevented the intermixing of the fish while permitting the free movement of infective stages through the water column. The donor and recipient compartments accounted for approximately one-third and two-thirds of the total tank volume, respectively, and contained 100 infected donor fish and 200 naïve recipient fish.

Throughout the exposure period, the fish were continuously monitored for feeding behavior, clinical signs, and mortality. Water exchange was minimized to preserve infective stages while maintaining optimal water quality conditions. Recipient fish were sampled on days 3, 7, and 14 postexposure, and gill smears were examined microscopically for parasite detection. The infection parameters were estimated via standard parasitological indices, including prevalence, mean intensity, and mean abundance^63^, which were calculated as follows:

$${\text{Prevalence }}\left( \% \right){\text{ }} = {\text{ }}\left( {{\mathrm{N}}_{i} /{\mathrm{N}}_{t} } \right){\text{ }} \times {\text{ 1}}00$$

$${\text{Mean intensity }} = {\text{ P}}_{t} /{\mathrm{N}}_{i}$$

$${\text{Mean abundance }} = {\text{ P}}_{t} /{\mathrm{N}}_{t}$$

where N_*i*_ = the number of infected fish, N_*t*_ = the total number of examined fish, and P_*t*_ = the total number of parasites recovered.

### Comparative therapeutic efficacy of *Y. schidigera* and fenbendazole baths following experimental monogenean challenge

Following the successful establishment of monogenean infection, the fish were randomly allocated into four experimental groups, each in triplicate. T1 served as the uninfected, untreated negative control; T2 served as the infected, untreated positive control; T3 served as the infected fish subjected to *Y. schidigera* bath treatment; and T4 served as the infected fish treated with Fenbendazole. A therapeutic bath was created under continuous aeration in accordance with established antiparasitic bath-treatment protocols^54,64^. The fish were exposed to the different treatments for 3 h daily over seven consecutive days. They were continuously monitored for behavioral changes, feeding activity, clinical manifestations, and mortality throughout the experimental period. At the end of the treatment regimen, gill smears were examined microscopically to determine parasite prevalence and intensity to evaluate treatment efficacy. The comparative therapeutic performance of the tested treatments was assessed based on parasite reduction and recovery under normal behavioral and physiological conditions, following standard parasitological procedures (Woo, 2006). Treatment efficacy (%) was calculated as follows:

$${\text{Efficacy }}\left( \% \right) = \left[ {\left( {{\mathrm{C}} - {\mathrm{T}}} \right)/{\mathrm{C}}} \right] \times {\mathrm{1}}00$$

where C = the mean parasite load in the control group and T = the mean parasite load in the treated group.

### Statistical analysis

Statistical analyses were performed via the Statistical Package for the Social Sciences (SPSS, version 25). Differences among mean water quality parameters, parasite intensity, and prevalence (%) were evaluated via two-way analysis of variance (ANOVA) according to Snedecor and Cochran^65^.

1 $$Y_{{ijk}} = \mu + F_{i} + M_{j} + (FM)_{{ij}} + \varepsilon _{{ijk}}$$

2 $${Y_{ijk}}=\mu +{F_i}+{M_j}+{\varepsilon _{ijk}}$$

where Yijkl represents the observed value (i.e., water temperature, pH, DO, total ammonia, salinity, and prevalence %), µ represents the overall mean, Fi represents the fixed effect of fish farms (i= farm 1 and farm 2), Mj represents the fixed effect of the month of four levels (February, March, April, and May), (FM)_ijk_ represents interaction, and ε_ijkl_ represents random error. The significance of differences between values was subsequently tested with Duncan’s multiple range tests^66^ as a post hoc test to detect particular differences between groups. The values are presented as the means ± standard errors (means ± S.E.M.s). Significance indicated in the figures and tables by an asterisk (*) was set at *p* < 0.05. Additionally, the associations among water quality parameters, prevalence percentages, and parasitic intensities were assessed using Pearson’s correlation matrix.

## Results

### Clinical and postmortem examination

**Fig. 1:**
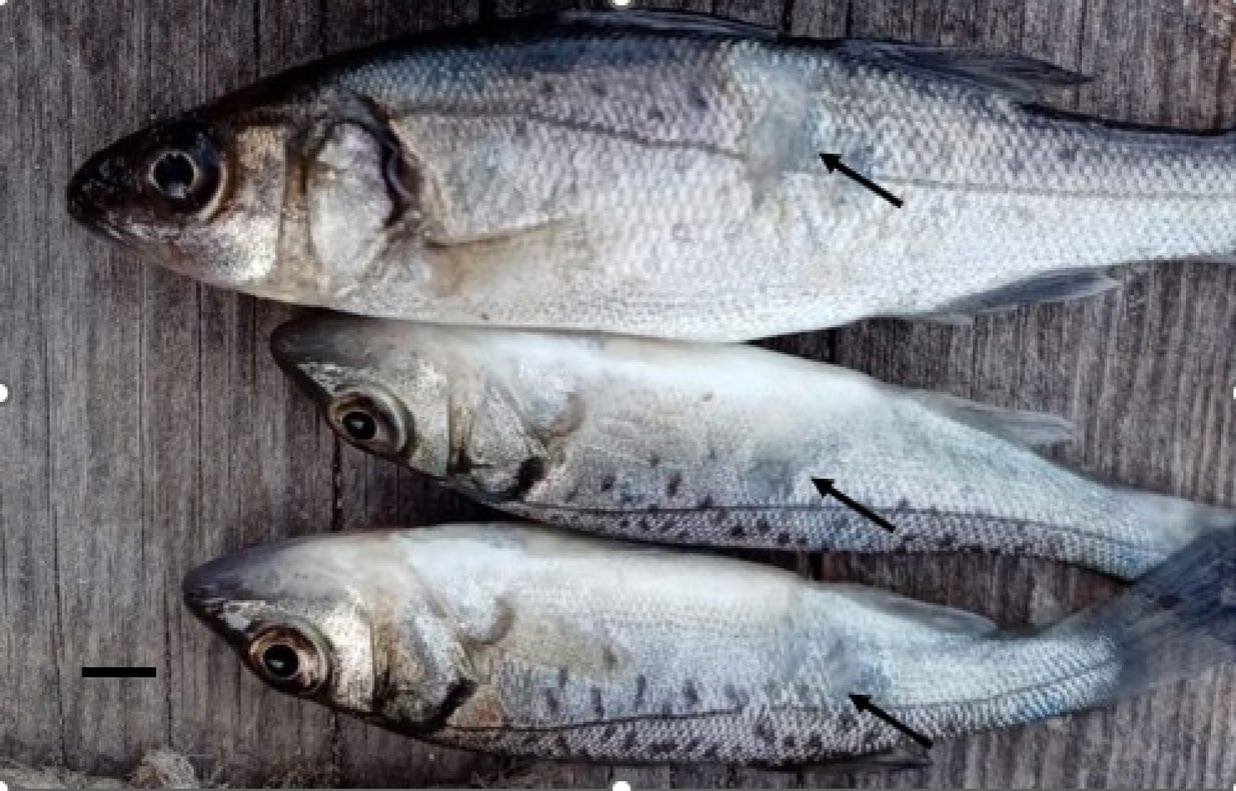
External lesions observed on the monogenean-infested *Dicentrarchus labrax*. The affected fish exhibited skin darkening, focal erosion, scale loss, and general debilitation. Scale bar = 1 cm.

During the early stage of monogenean infestation, *D. labrax* exhibited no apparent clinical abnormalities under low parasite burdens. As the infection progressed, the affected fish presented reduced feed intake, lethargy, diminished swimming activity, anorexia, and occasional aggregation near the water surface. With increasing parasite intensity, more pronounced clinical manifestations, including excessive mucus secretion, skin darkening, respiratory distress, and flashing behavior, accompanied by focal skin erosion, mild scale loss, and scale detachment, became evident (Fig. 1). In the advanced stages of infestation, the fish became severely debilitated, exhibiting pale gills, marked skin discoloration, increased opercular movements, piping behavior, and persistent surface aggregation, frequently culminating in mortality.

### Parasitological examination

**Fig. 2:**
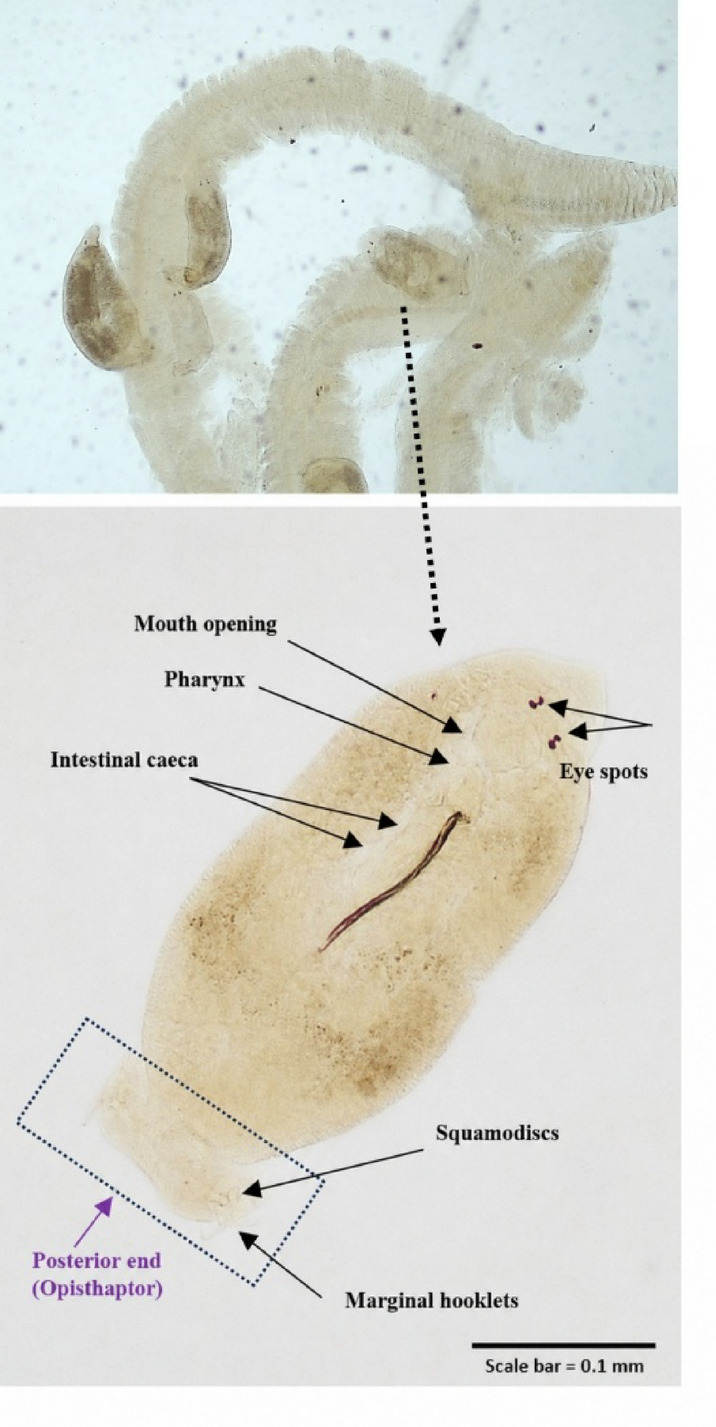
Gill smears collected from European sea bass (*Dicentrarchus labrax*) showing the monogenean parasite *Diplectanum laubieri*. The opisthaptor bears two well-developed squamodiscs (dorsal and ventral), with small marginal hooklets distributed along the haptoral margin. The anterior region (prohaptor) shows two pairs of eyespots and a distinct, rounded, spherical pharynx. Scale bar = 0.1 mm.

Microscopic examination revealed an elongate-oval monogenean parasite with a slightly tapered anterior end and a comparatively broader posterior region. Paired longitudinal intestinal caeca were observed as internal structures consistent with monogenean morphology. The posterior attachment organ (opisthaptor) consisted of two well-developed squamodiscs (dorsal and ventral), with small marginal hooklets distributed along the haptoral margin. The anterior region (prohaptor) possessed two pairs of eyespots and a distinct, rounded, spherical pharynx. These morphological characteristics are consistent with those of *Diplectanum laubieri* (Monogenea: Diplectanidae) (Fig. 2).

### Prevalence of monogenean infestation

The monogenean prevalence varied significantly among farms and sampling months (Table 1). Farm 2 had a significantly higher prevalence (75.00 ± 6.87%) than Farm 1 (65.56 ± 7.51%) (*p* < 0.05). Temporally, the highest prevalence was recorded in February (87.78 ± 3.33%), followed by a progressive decline in March (71.11 ± 2.22%) and April, when the lowest prevalence was observed (53.34 ± 4.45%). The prevalence subsequently increased again in May (68.89 ± 8.89%).

**Table 1:** Monogenean prevalence in farmed *Dicentrarchus labrax* from farms under study in Egypt.

Variables	Levels	Prevalence (Mean ± S.E.)
**Farm**	Farm 1	65.56^b^±7.51
	Farm 2	75.00^a^±6.87
**Month**	February	87.78^a^±3.33
	March	71.11^b^±2.22
	April	53.34^c^±4.45
	May	68.89^b^±8.89

Means bearing different superscript letters within each independent variable differ significantly at *p* < 0.05.

**Fig. 3:**
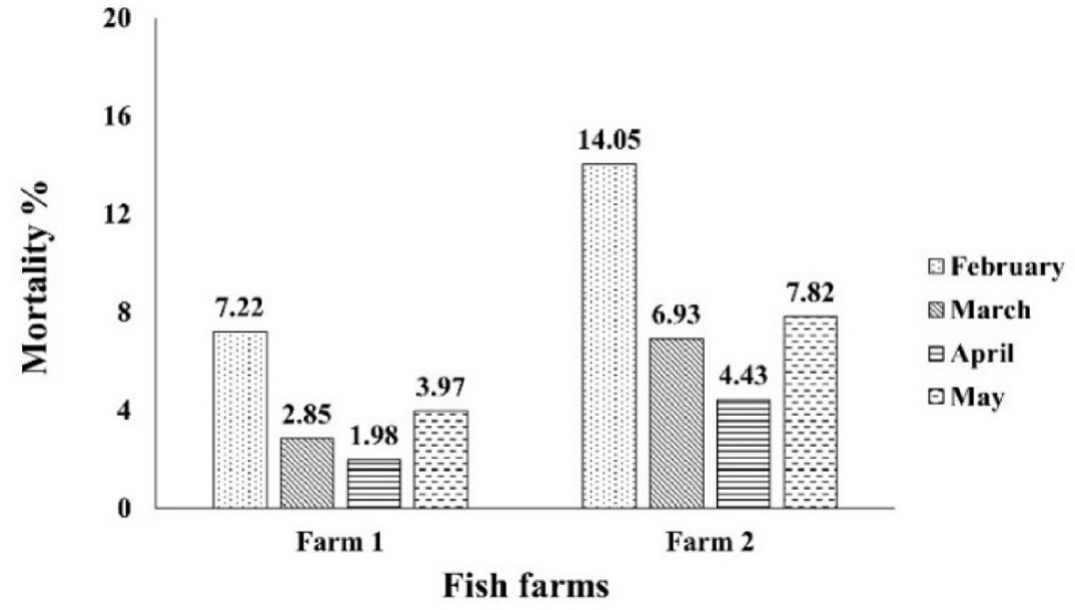
Monthly mortality (%) of monogenean-infested *Dicentrarchus labrax* in Farm 1 and Farm 2 during the sampling period (February–May 2024).

The mortality rates of *D. labrax* also markedly varied between the two farms throughout the study period. Farm 2 consistently recorded higher mortality rates than Farm 1 across all the sampling months. The highest mortality rate for Farm 2 was observed in February (14.05%), followed by a decline in March and April, before increasing again in May (7.82%). A similar trend was observed for Farm 1, where mortality peaked in February (7.22%), declined to its lowest level in April (1.98%), and then slightly increased in May (3.97%) (Fig. 3).

### Monogenean intensity

**Fig. 4:**
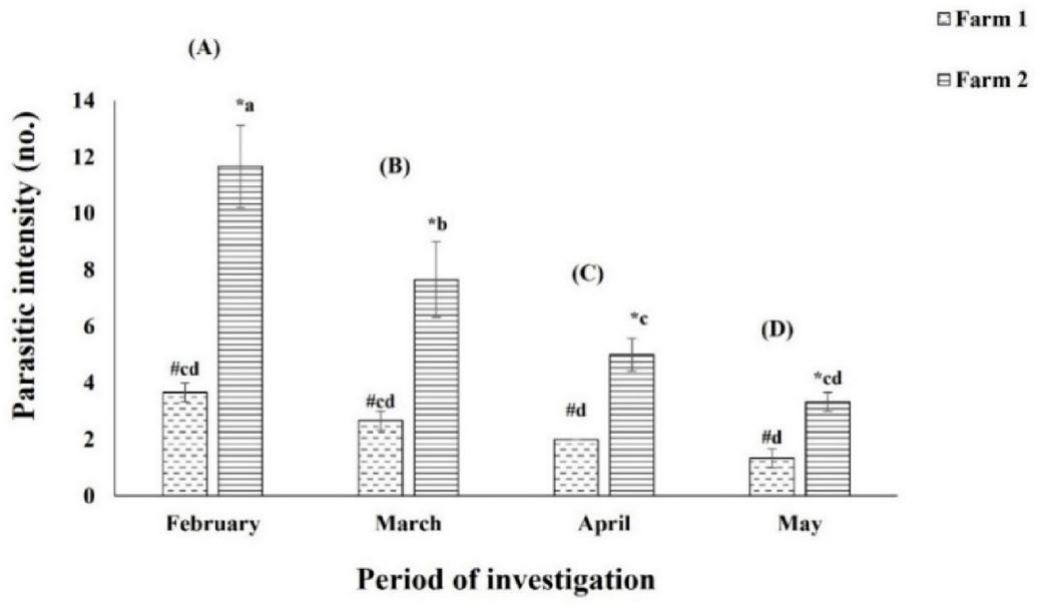
Parasitic intensity of monogenean infestations in *Dicentrarchus labrax* collected from two fish farms during the study period (February–May). The data are presented as the means ± SEs. Different lowercase letters indicate significant differences within each farm across sampling periods, whereas uppercase letters indicate significant differences among months irrespective of farm. Asterisks (*) and hash symbols (#) denote significant differences between the two farms at each sampling point.

The parasitic intensity of *D. labrax* infested with monogeneans differed significantly between the two farms throughout the study period. Farm 2 consistently recorded higher parasitic intensity than Farm 1 did, with the highest level observed in February (11.9 parasites/fish), followed by a decline in May (3.3 parasites/fish). Similarly, Farm 1 presented lower infection levels, decreasing from 3.7 parasites/fish in February to 1.3 parasites/fish in May (Fig. 4).

### Molecular identification

**Fig. 5:**
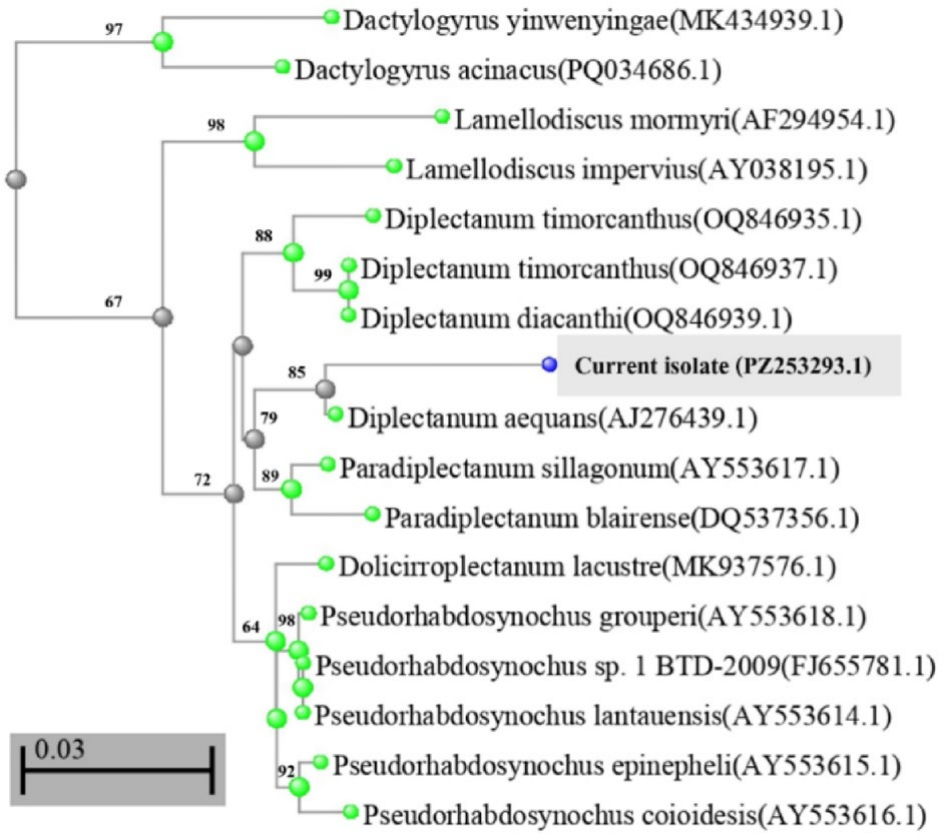
A neighbor‒joining (NJ) phylogenetic tree was constructed based on alignments of rRNA and ITS sequences. The NJ method is based on the Tamura‒Nei model within MEGA 11. Bootstrap values are clearly labeled at each node of the tree.

The sequencing data revealed a unique partial sequence of 502 bp. Nucleotide BLASTn analysis of the sequence yielded approximately 100 homologous sequences, with most hits being partial *18 S rRNA* sequences from diverse taxa of the monogenean flatworms. After excluding the repetitive hits from the same taxa and those with lower homologies (< 80% identity and query coverage), 17 hits were included in the subsequent homology and phylogenetic analysis, including the current isolate. The 17 most homologous sequences comprised partial sequences of the small subunit rRNA and ITS genes from monogenean genera as diverse as *Diplectanum*, *Dolicirroplectanum*,* Pseudorhabdosynochus*,* Lamellodiscus*, and *Dactylogyrus*. Among the top hits (> 90% nucleotide identity and query coverage) were partial rRNA sequences of Diplectanid species such as *Diplectanum aequans*, *D. timorcanthus*, and *D. diacanthi*. The partial 502-nucleotide sequence of the current study was deposited into GenBank under the accession number PZ253293.1. NJ-based phylogenetic analysis of *18 S rRNA* sequences separated monogenean parasites into two distinct clades (Fig. 5). The first clade has two branches representing the species *Dactylogyrus yinwenyingae* and *D. acinacus*, with significant bootstrap support of 97% between them. The second clade, which is separated from the Dactylogyrid clade by 67% bootstrap support, is divided into two main clades: one that includes the two species *Lamellodiscus*, while the remaining monogenean genera reside in the second-largest clade of the phylogenetic tree (Fig. 5). Species of diplectanids are clustered into two small clades, with *D. timorcanthus* and *D. diacanthi* in a single clade, whereas the current isolate is grouped with *D. aequans* in the second clade, with bootstrap support confidence of 85. The two species of Paradiplectanum branch from the same node, whereas the single species of *Dolicirroplectanum*,* D. lacustre*, is singly branched. The five isolates of *Pseudorhabdosynochus* clustered into two clades with strong bootstrap support. Phylogenetic analysis, together with the strong evolutionary relatedness and clustering with other *Diplectanum* species, further supports the taxonomic assignment of the present isolate within the genus *Diplectanum*. Integrating molecular and morphometric evidence has additionally reinforced the putative identification of the monogenean isolate as *Diplectanum laubieri*.

### Water physicochemical parameters

**Fig. 6:**
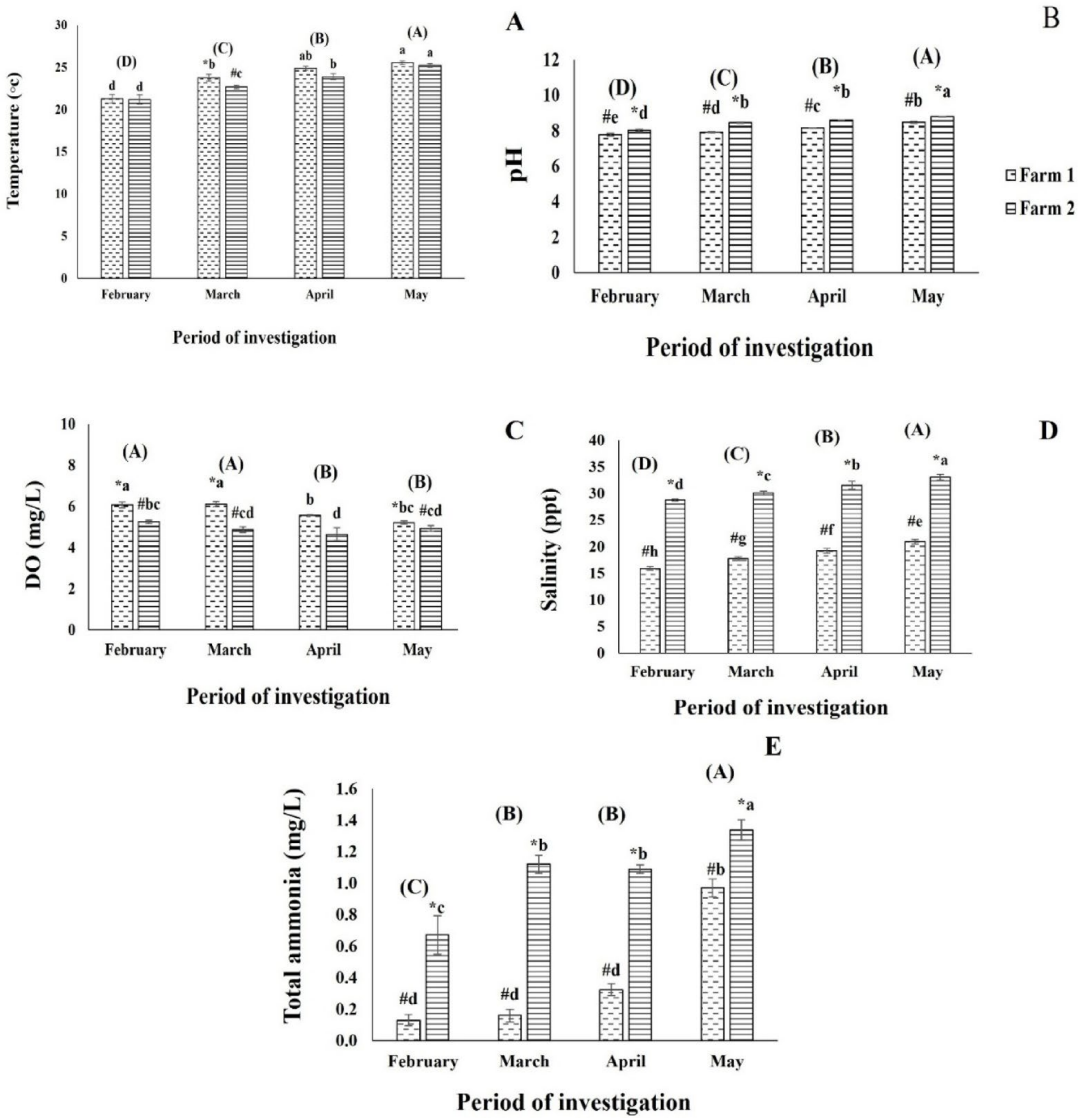
Physicochemical water parameters, including water temperature (°C) (**A**), pH (**B**), dissolved oxygen (DO, mg/L) (**C**), salinity (ppt) (**D**), and total ammonia (mg/L) (**E**), were measured in the two fish farms during the study period (February–May). The data are presented as the means ± SEs. Different lowercase letters indicate significant differences within each farm across sampling periods, whereas uppercase letters indicate significant differences among months regardless of farm. Asterisks (*) and hash symbols (#) indicate significant differences between the two farms at each sampling point.

Throughout the study period, all measured water quality parameters exhibited significant temporal and spatial variation between the two fish farms (Fig. 6A-E). The water temperature increased progressively from February to May, reaching its peak in May (25.40 ± 0.16 °C), with Farm 1 showing a slightly higher overall mean (23.88 ± 0.51 °C) than Farm 2 (23.25 ± 0.48 °C). A similar monthly trend was observed for pH, which increased from February to May and was consistently greater in Farm 2 (8.46 ± 0.09) than in Farm 1 (8.08 ± 0.08), peaking in May (8.63 ± 0.07). Dissolved oxygen (DO) showed an inverse pattern, declining over time and remaining significantly greater in Farm 1 (5.74 ± 0.12 mg/L) than in Farm 2 (4.93 ± 0.10 mg/L), with the highest values recorded in February–March and the lowest in April–May. Salinity showed a strong spatial contrast, being markedly greater at Farm 2 (30.87 ± 0.52) than at Farm 1 (18.47 ± 0.58) and increasing gradually from February to May. In contrast, total ammonia exhibited a progressive increase across months on both farms, reaching maximum levels in May (1.15 ± 0.09 mg/L), with consistently higher concentrations in Farm 2 (1.05 ± 0.08 mg/L) than in Farm 1 (0.40 ± 0.10 mg/L), indicating a deterioration in water quality over time, particularly in Farm 2.

### Correlation between physicochemical parameters and parasite prevalence

Table 2 presents the correlation matrix that describes the relationships between water quality parameters and the prevalence of parasitic infection. The prevalence of infection exhibited a significant negative correlation with water temperature (*r* = -0.736, *p* < 0.05), whereas no statistically significant correlations were detected with pH, dissolved oxygen, total ammonia, or salinity. With respect to the interrelationships among the physicochemical variables, temperature had a positive but nonsignificant association with pH (*r* = 0.648, *p* > 0.05). In contrast, pH was positively correlated with total ammonia (*r* = 0.936, *p* < 0.01) and salinity (*r* = 0.755, *p* < 0.05). Dissolved oxygen was inversely correlated with pH, total ammonia, and salinity. Furthermore, total ammonia showed a strong positive correlation with salinity (*r* = 0.876, *p* < 0.01).

**Table 2:** Correlation matrix between water quality parameters and parasite prevalence.

Parameters	Parasitic prevalence	Temperature	pH	DO	Total Ammonia	Salinity
Temperature	-0.736*	1	0.648	-0.273	0.377	0.068
pH	-0.321	0.648	1	-0.869**	0.936**	0 0.755*
DO	0.151	-0.273	-0.869**	1	-0.933**	-0.899**
Total ammonia	-0.031	0.377	0.936**	-0.933**	1	0.876**
Salinity	0.174	0.068	0 0.755*	-0.899**	0.876**	1

The values represent Pearson’s correlation coefficients (r). ** Correlation is significant at *p* < 0.01 (2-tailed). * Correlation is significant at *p* < 0.05 (2-tailed).

### Phytochemical analysis of *Y. schidigera* extract

Quantitative analysis of *Y. schidigera* extract revealed a total phenolic content (TPC) of 12.57 mg gallic acid equivalents (GAE)/g dry plant material, corresponding to 2.51 mg GAE/mL crude extract. The total flavonoid content (TFC) was 2.40 mg quercetin equivalents (QE)/g dry material, equivalent to 0.48 mg QE/mL of extract. Both parameters were determined via external standard calibration curves of gallic acid and quercetin.

### Determination of safe concentrations of *Y. schidigera* and Fenbendazole in a bath

A toxicity assessment conducted to establish safe bath concentrations of *Y. schidigera* and fenbendazole is summarized in Table 3. Fish exposed to *Y. schidigera* at concentrations ranging from 50 to 200 µg/L exhibited 100% survival, with no mortality recorded. At 300 µg/L, mild toxic effects resulted in 16.7% mortality, which increased substantially to 63.3% at 400 µg/L. Based on these findings, 200 µg/L was established as the highest nonlethal concentration, while 150 µg/L was considered the most appropriate safe therapeutic dose for subsequent bath treatments. Similarly, fenbendazole baths were well tolerated at concentrations up to 30 µg/L, with no observed mortality. However, mortality increased to 20% at 40 µg/L and further increased to 46.7% at 50 µg/L, indicating clear dose-dependent toxicity. Accordingly, 20 µg/L was determined as the optimal safe therapeutic concentration for fenbendazole under the present experimental conditions.

**Table 3:** Toxicity assay of *Yucca schidigera* and fenbendazole bath therapy in *Dicentrarchus labrax*.

Therapeutic agent	Fish No.	Concentration (µg/L)	Dead fish No.	Mortality %	Safety assessment
***Yucca schidigera***	30	50	-	0	Safe
	30	100	-	0	Safe
	30	200	-	0	Safe
	30	300	5	16.67	Mildly toxic
	30	400	19	63.33	Highly toxic
Fenbendazole	30	10	-	0	Safe
	30	20	-	0	Safe
	30	30	-	0	Safe
	30	40	6	20	Mildly toxic
	30	50	14	46.67	Highly toxic

### Establishment of experimental monogenean infection via cohabitation

The progression of monogenean infection in *D. labrax* under cohabitation challenge is presented in Table 4. The model successfully established a time-dependent increase in both infection prevalence and intensity in recipient fish. Before exposure (day 0), all the fish were confirmed to be free of monogenean infection. By day 3 postexposure, a low-level infection was detected (prevalence: 18.3%; intensity: 1.2 ± 0.4 parasites per microscopic field) without observable clinical signs. On day 7, infection increased markedly (62.5% prevalence; 3.8 ± 1.6 parasites per microscopic field), accompanied by reduced feeding activity. By day 14, a heavy infection was established, characterized by high prevalence (~ 90.0%) and elevated parasite intensity (8.5 ± 1.9 parasites per microscopic field), together with pronounced clinical manifestations, including respiratory distress, increased opercular activity, loss of equilibrium, and erratic swimming behavior.

**Table 4:** Progression of monogenean infection in *Dicentrarchus labrax* under cohabitation challenge.

Exposure day	Examined fish No.	Prevalence %	Mean intensity (No. parasites /Microscopic field)	Clinical signs
0	30	0	-	No infection
3	30	18.3	1.2 ± 0.4	Apparent normal
7	30	62.5	3.8 ± 1.6	Mild
14	30	90.0	8.5 ± 1.9	Severe

### Comparative therapeutic efficacy of *Yucca schidigera* and Fenbendazole

A comparative evaluation of parasite reduction, clinical recovery, and mortality following bath treatments with *Y. schidigera* and fenbendazole in monogenean-infected *D. labrax* is presented in Table 5. Both treatments significantly reduced monogenean infection compared with the infection control (T2: 100% prevalence, 9.2 ± 2.1 parasites/field, 26.67% mortality). Compared with fenbendazole (T4), *Yucca schidigera* (T3) showed greater therapeutic efficacy, reducing the prevalence to 20%, reducing the parasite intensity to 1.8 ± 0.6 parasites/field, and achieving 80.4% parasite reduction with 6.67% mortality, when compared with fenbendazole, which reduced the prevalence to 30%, the intensity to 2.9 ± 0.9, and achieving a 68.5% reduction with 10.0% mortality.

**Table 5:** Comparative therapeutic efficacy of *Y. schidigera* and Fenbendazole against monogenean infection in *Dicentrarchus labrax*.

Efficacy patterns	T1	T2	T3	T4
Number of fish (n)	30	30	30	30
Infected fish (n)	0	30	6	9
Prevalence (%)	0	100	20.0	30.0
Mean parasite intensity (parasites/field)	0	9.2 ± 2.1	1.8 ± 0.6	2.9 ± 0.9
Parasite reduction efficacy (%)	-	0	80.4	68.5
Mortality (n)	0	8	2	3
Mortality (%)	0	26.67	6.67	10.0

T1 (uninfected negative control), T2 (infected untreated positive control), T3 (infected fish treated with a *Y. schidigera* bath), and T4 (infected fish treated with a fenbendazole bath), with 30 fish per group distributed across triplicate tanks (*n* = 3).

## Discussion

Monogenean infestations remain among the most challenging parasitic diseases affecting marine aquaculture owing to their direct life cycle, rapid transmission, and marked pathogenicity under intensive farming conditions. In the present study, the progressive deterioration in the health status of farmed *Dicentrarchus labrax* infected with *Diplectanum laubieri* reflects the substantial physiological burden imposed by diplectanid monogeneans on cultured seabass. The observed behavioral abnormalities are characteristic stress responses commonly associated with gill parasitism and respiratory compromise in marine fishes. Similar clinical manifestations have previously been reported in seabass infected with *Diplectanum* spp. and other monogeneans, where parasite attachment to the gill epithelium resulted in impaired respiration, irritation, and progressive debilitation^67–69^.

In *D. labrax* infected with monogeneans, the initial clinical signs included reduced feed intake, lethargy, and diminished swimming activity. As infestation intensity increased, these signs progressed to more severe manifestations, including respiratory distress, excessive mucus production, skin darkening, flashing behavior, focal skin erosions, and persistent aggregation near the water surface. Similar clinical and behavioral changes have been previously reported in European seabass naturally infected with *Diplectanum* spp.^70,71^. These manifestations could be associated with the mechanical irritation, attachment activity, and feeding behavior of monogeneans on branchial tissues, resulting in epithelial damage, mucous cell hyperactivity, and inflammatory degeneration, which consequently impair branchial gas exchange and osmoregulatory functions^37,72^.

Morphological identification of the recovered parasite as *D. laubieri* was supported by the characteristic presence of paired squamodiscs, marginal hooklets, eyespots, and a well-developed opisthaptor. These diagnostic features are consistent with previous morphological descriptions of diplectanid monogeneans infecting marine teleosts^19,73^. The highly specialized attachment apparatus of diplectanids represents a major pathogenic determinant because continuous anchoring to the gill lamellae causes persistent mechanical trauma, epithelial erosion, and localized hemorrhage. Similar observations were reported by Dezfuli et al.^37^, who reported severe pathological alterations in the gills of seabass infected with *D. aequans*.

The molecular identification of monogenean flatworms has become a fundamental component of integrative taxonomy, providing essential support for traditional morphometric approaches. In the present study, the molecular characterization of the isolate from *D. labrax* was based on a 502 bp fragment of the *18 S rRNA* gene (GenBank: PZ253293.1), which supports its assignment to Diplectanum and its tentative identification as *D. laubieri*. BLAST analysis revealed the highest sequence similarity (96.18%) with *Diplectanum aequans* (accession number: AJ276439.1). Both *D. laubieri* and *D. aequans* are recognized co-occurring parasites of *D. labrax* and are known to occupy distinct microhabitats on the gills^10,12^. The close genetic similarity between these species, together with their clustering in the phylogenetic tree (85% bootstrap support), reflects their strong evolutionary relatedness and shared host associations. In contrast, lower sequence identities were observed with the recently described Australian species *Diplectanum timorcanthus* (92.99%) and *Diplectanum diacanthi* (93.12%), both reported from *Protonibea diacanthus* in northern Australian waters^44^. This reduced homology highlights genetic divergence within the genus, likely driven by geographic isolation and host-driven evolutionary processes. Phylogenetic reconstruction via the neighbor‒joining method clearly resolved the Diplectanidae into distinct clades.

The present isolate clustered within a strongly supported *Diplectanum* clade alongside *D. aequans*, confirming its generic placement. The clear separation of *Diplectanum* from genera such as *Lamellodiscus* and *Pseudorhabdosynochus* aligns with previous molecular phylogenetic studies^74,75^. Notably, *Lamellodiscus mormyri* and *L. impervius* formed distinct lineages outside the main *Diplectanum* cluster, supporting the utility of the *18 S rRNA* marker for resolving higher-level phylogenetic relationships within Diplectanidae. Similarly, the distinct clustering of Pseudorhabdosynochus species highlights the molecular diversity within the family and supports the application of integrative taxonomic approaches in monogenean systematics. The present isolate clustered within the *Diplectanum* lineage and showed closest affinity to *D. aequans*, confirming its placement within the genus. However, given the conserved nature of the *18 S rRNA* gene and the limited sequence length analyzed, species-level identification should be interpreted cautiously. Therefore, the assignment to *Diplectanum laubieri* is based primarily on the concordance between morphological characteristics and molecular evidence, while additional markers such as ITS1, ITS2, 28 S rDNA, or mitochondrial COI would be valuable for definitive species confirmation.

The significantly greater monogenean infestation in Farm 2 suggests that environmental stress conditions may increase the susceptibility of fish to monogenean infections due to immunosuppression and impaired mucosal defenses. The lower dissolved oxygen concentrations and higher ammonia and salinity levels recorded in Farm 2 may therefore have contributed to enhanced host susceptibility and facilitated parasite establishment. Similar associations between deteriorated water quality and increased parasitic intensity, which may promote monogenean proliferation and disease outbreaks, have been documented in various marine fishes^76^. Parasitic infections are also considered potential bioindicators of ecosystem health, emphasizing the influence of farming practices on parasite transmission and disease dynamics^77,78^.

Monthly variation plays a significant role in shaping monogenean transmission dynamics and establishment. The higher prevalence and infection intensity observed during colder months suggest that reduced temperatures may increase parasite persistence and transmission. Temperature is a key ecological driver of monogenean biology, influencing egg development, hatching rates, larval survival, and the transmission efficiency of *Diplectanum* spp. infecting European sea bass^79^. Similarly, Ozer et al.^80^ and Li et al.^81^ reported temporal fluctuations in monogenean prevalence associated with variations in environmental temperature. Lower winter temperatures may prolong larval viability and increase attachment success, thereby increasing infection pressure during colder periods. In addition, elevated ammonia levels can damage branchial tissues and suppress immune responses, increasing host susceptibility to monogenean infections^82^. The inverse relationship between dissolved oxygen and ammonia observed in Farm 2 further indicates progressive environmental deterioration, which may intensify the pathological impact of gill parasitism by exacerbating respiratory stress and reducing host tolerance. Comparable mortality patterns associated with diplectanid infections have been documented in Mediterranean and Black Sea cultured seabass^37^, underscoring the considerable health and economic implications of these parasites in intensive aquaculture systems. Moreover, the higher parasite intensity recorded in February and its persistence through March and April likely reflect the seasonal modulation of diplectanid transmission dynamics. These patterns suggest that colder conditions may sustain transmission, whereas subsequent increases in temperature may influence parasite development, reproductive activity, and overall transmission potential, ultimately contributing to elevated infection levels in cultured seabass^83^.

The imprudent and repeated use of chemotherapeutic agents in aquaculture has raised major concerns regarding antimicrobial resistance, reduced treatment efficacy, drug residues in fish tissues, environmental contamination, and adverse effects on nontarget organisms^84^. Consequently, considerable attention has shifted toward eco-friendly alternatives, particularly functional feed additives and phytobiotic compounds, including probiotics, prebiotics, synbiotics, and medicinal plant extracts^85–87^. Among these phytobiotics, *Y. schidigera* has gained increasing interest because of its beneficial effects on growth performance, feed utilization, immune responses, antioxidant status, and water quality across several fish species^88,89^. Accordingly, the present study evaluated the antiparasitic efficacy and safety profile of *Y. schidigera* bath treatment in comparison with fenbendazole for controlling monogenean infestation in cultured *D. labrax*. Phytochemical analysis of *Y. schidigera* extract revealed considerable levels of total phenolics (12.57 mg GAE/g; 2.51 mg GAE/mL) and total flavonoids (2.40 mg QE/g; 0.48 mg QE/mL), which are bioactive compounds widely recognized for their antioxidant, anti-inflammatory, antimicrobial, and antiparasitic properties^90,91^.

Given the limited available data regarding the safety assessment and dose optimization of *Y. schidigera* and fenbendazole in European seabass, the present findings were interpreted in relation to previously established safety models and the therapeutic ranges reported for other phytobiotic and antiparasitic agents in fish. *Y. schidigera* bath treatment exhibited a wider safety margin, being well tolerated up to 200 µg/L, whereas fenbendazole demonstrated acceptable safety only at the lower concentration of 20 µg/L. Previous studies have reported the general tolerability of *Y. schidigera* under aquatic exposure conditions. In Nile tilapia, continuous exposure to 1.0 g/L *Y. schidigera* enhanced antioxidant defenses^89^. In European sea bass juveniles, prolonged exposure to 0.75 mg/L improved hematological and immune indices over 93 days^34,92^. Similarly, repeated supplementation at 8 mg/L every two days alleviated hypoxia-induced stress in Nile tilapia while improving growth performance and biochemical status^93^.

Short-term herbal bath treatments administered over a broad range of concentrations have also consistently demonstrated significant antiparasitic efficacy against monogenean infestations across diverse fish species. An aqueous extract of *Allium sativum* at 7.5–12.5 mL/L for 1 h effectively controlled *Gyrodactylus turnbulli* in guppies^94^, whereas an ethyl acetate extract of *Euphorbia fischerana* at 14 mg/L for 48 h reduced *Dactylogyrus vastator* infestation in goldfish^95^. Similarly, *Camellia sinensis* bath treatment at 0.3–0.9% for 1–5 min effectively controlled *Neobenedenia* spp. in salmonids^96^, whereas the addition of the methanolic extract of *Melia azedarach* at 381 mg/L for 48 h significantly reduced *Gyrodactylus kobayashii* infection in Goldfish^97^. In addition, extracts of *Bupleurum chinense*, *Polygonum multiflorum*, *Dioscorea collettii*, and *Citrus medica* administered at concentrations of 6.9–125 mg/L exhibited potent activity against *Dactylogyrus intermedius*^98^. Other medicinal plants, including *Cinnamomum cassia*, *Lindera aggregata*, *Pseudolarix kaempferi*, *Dryopteris crassirhizoma*, *Kochia scoparia*, *Polygala tenuifolia*, *Caesalpinia sappan*, *Lysimachia christinae*, *Clematis chinensis*, *Artemisia argyi*, and *Eupatorium fortunei*, also demonstrated marked antiparasitic efficacy against *D. intermedius* at concentrations ranging from 22.97 to 500 mg/L following 48 h of bath exposure^99,100^. The observed variation in the safe and effective concentrations of *Y. schidigera* among studies is likely attributable to differences in extract preparation, phytochemical composition, fish species, exposure duration, dosage regimen, parasite burden, and environmental conditions. Therefore, the relatively high safe concentration observed in the present study may be related to the short-term exposure strategy combined with water renewal conditions, which may have reduced compound accumulation and associated physiological stress in the fish.

Fenbendazole exhibited a relatively narrow therapeutic safety margin and a pronounced time- and dose-dependent toxicity profile, being tolerated up to 30 µg/L without mortality, whereas mortality increased to 20% and 46.7% at 40 and 50 µg/L, respectively. Kolarova et al.^101^ reported that fenbendazole bath treatment (25 mg/L; 2 × 12 h with a 24 h interval) reduced parasite intensity by more than 90%. The lower safe concentration established in the present study likely reflects differences in fish species, experimental design, exposure regimen, treatment duration, and fenbendazole formulation. As these factors can substantially influence drug toxicity and therapeutic tolerance, direct comparison between the two studies is not appropriate. Consequently, the safe therapeutic concentration of fenbendazole should be established for each target species under the specific treatment conditions. Oral administration of 50 mg/kg body weight for three consecutive days did not produce observable adverse effects, including mortality or reduced feed intake^102^. Dong et al. (2025) reported a time- and dose-dependent toxic response, where exposure to 0.06 mg/L resulted in low mortality within the first 48 h, followed by a marked increase reaching 100% after 96 h of exposure. These differences in safety and efficacy across studies likely reflect species-specific tolerance, treatment methods (bath versus oral administration), drug concentrations, exposure durations, and environmental conditions.

Comparative therapeutic evaluation revealed greater antiparasitic efficacy of *Yucca schidigera* than fenbendazole against monogenean infection in *D. labrax*. *Y. schidigera* reduced the prevalence to 20%, decreased the parasite intensity to 1.8 ± 0.6 parasites/field, achieved 80.4% parasite reduction, and resulted in lower mortality (6.67%). In contrast, fenbendazole (T4) showed comparatively lower efficacy, with a higher prevalence (30%), greater parasite intensity (2.9 ± 0.9 parasites/field), lower parasite reduction (68.5%), and increased mortality (10.0%). The principal antiparasitic mechanism of saponins involves alterations in parasite membrane permeability, resulting in membrane disruption and impaired parasite survival. In parallel, phenolic compounds exert antioxidant activity through scavenging reactive oxygen species and reducing parasite-induced oxidative stress, thereby supporting tissue recovery and host resilience^103^. Additionally, the immunostimulatory effects of *Y. schidigera* may enhance fish defense mechanisms against parasitic infestation. Both dietary and waterborne applications have been reported to stimulate innate and humoral immune responses in fish, while phenolic hydroxyl groups contribute to antioxidant defense mechanisms. For example, dietary supplementation with *Y. schidigera* increased the total antioxidant capacity of mirror carp^35^; elevated SOD, CAT, and GPx activities; reduced lipid peroxidation in Nile tilapia (Abdel-Tawwab et al., 2021); and improved antioxidant responses in olive flounder^104^.

Water quality improvement is another beneficial effect of *Y. schidigera*, achieved through reducing ammonia accumulation and associated environmental stress, thereby indirectly enhancing disease resistance in cultured Nile tilapia and European seabass juveniles^105^, as well as striped catfish^106^. This effect is likely attributable to the surface-active properties of steroidal saponins and glycol compounds, which may reduce ammonia accumulation in the aquatic environment. The consequent decrease in water pH can lower concentrations of toxic unionized ammonia (NH₃), thereby mitigating environmental conditions that favor parasite establishment, survival, and proliferation^107^. Collectively, these direct and indirect actions support the potential application of *Y. schidigera* as a safer and eco-friendly phytobiotic alternative because of its biodegradability, relatively low toxicity, and broad safety margin^108,109^. In contrast, the reduced efficacy of fenbendazole may be related to the mode of action of benzimidazoles, which impair parasite energy metabolism through disruption of microtubule formation, leading to progressive energy depletion and eventual parasite death^110^. Because this process is cumulative and time-dependent, benzimidazoles may exert a delayed anthelmintic effect that persists after drug removal. This finding supports the feasibility of short-duration exposure strategies, as also reported for febantel, where antiparasitic activity continued during the posttreatment period in drug-free water^111^.

## Conclusion

The present study demonstrated that the pathogenicity of *D. laubieri* is governed not only by parasite presence but also by a multifactorial interplay among parasite burden, host condition, environmental quality, and seasonal variability. The highest prevalence and infection intensity were recorded on the farm characterized by elevated salinity, pH, and ammonia concentrations, coupled with reduced dissolved oxygen levels. Morphological examination and molecular characterization supported the tentative identification of the isolate as *D. laubieri*. Correlation analysis further indicated that parasite prevalence was significantly associated with water temperature and salinity. Moreover, compared with fenbendazole, *Y. schidigera* exhibited higher efficacy under the tested conditions in reducing the parasite load and associated mortality under the tested conditions. Collectively, these findings underscore the critical role of environmental water quality in modulating monogenean infection dynamics and support the potential application of *Y. schidigera* as a complementary, environmentally compatible approach for parasite management in marine aquaculture systems.

### Study limitations

The parasite intensity was estimated as the average number of parasites per microscopic field rather than by complete enumeration of parasites on all gill arches. Although this approach provides a practical estimate of infection intensity, whole-gill parasite counts would yield a more comprehensive assessment of parasite burden and improve comparability among future studies.

## Data Availability

The datasets generated and/or analyzed during the current study are available in the GenBank repository under accession number PZ253293.1 and can be accessed at https://www.ncbi.nlm.nih.gov/nuccore/3263490407.
